# Malignant Local Seeding in Procedure Tracts of Pleural Mesothelioma: Incidence and Novel Risk Factors in 308 Patients

**DOI:** 10.3390/cancers17172786

**Published:** 2025-08-26

**Authors:** Moshe Lapidot, Emanuele Mazzola, Raphael Bueno

**Affiliations:** 1 Division of Thoracic Surgery, Lung Center and International Mesothelioma Program, Brigham and Women’s Hospital and Harvard Medical School, Boston, MA 02115, USA; rbueno@bwh.harvard.edu; 2 Division of Thoracic Surgery, Galilee Medical Center, Nahariya 2210001, Israel; 3 Dana Farber Cancer Institute, T.H Chan School of Public Health, Boston, Harvard Medical School, Boston, MA 02215, USA; mazzoloa@ds.dfci.harvard.edu

**Keywords:** pleural mesothelioma, procedure tracts, pleurectomy decortication, video-assisted thoracic surgery

## Abstract

**Background/Objectives**: Unlike other thoracic malignancies, seeding malignant cells along surgical tracts is a known complication of invasive diagnostic or therapeutic procedures for pleural mesothelioma (PM). We report the tract dissemination rate and risk factors in 308 consecutive patients treated over 9 years in a single institution who underwent pleurectomy decortication (PD). **Methods**: Clinical and outcome data were reviewed. Fisher’s exact test, Kaplan–Meier estimators, and log-rank tests were used to identify significant risk factors for surgical tract dissemination and to compare overall survival. **Results**: There were 233 males (75.6%), 187 right-sided operations (61%), 190 (61.7%) epithelioid histology cases, and the median age was 69 (29–84). During the study, malignant cell dissemination in resected surgical tracts was diagnosed in 69 (22.4%) patients. The dissemination rates in epithelioid, biphasic, and sarcomatoid tumors were 24.7%, 20.4%, and 0%, respectively. Disseminated malignant surgical tract was associated with advanced nodal status (*p* = 0.001), advanced staging by the American Joint Committee on Cancer (AJCC 8th edition, *p* = 0.03), female sex (0.02), side of surgery (*p* = 0.03), and the number of video-assisted thoracoscopic surgery (VATS) ports (*p* = 0.003). In epithelioid mesothelioma, the median survival from diagnosis was 19.7 months in patients with tract seeding versus 36.3 months in patients without seeding (hazard ratio, 1.9; *p* = 0.001). **Conclusions**: Procedure tract dissemination occurs in almost every fourth patient with pleural mesothelioma and is associated with shorter overall survival in the epithelioid subtype.

## Simple Summary

Procedure tract malignant dissemination is exceedingly rare in thoracic malignancies, with the exception of pleural mesothelioma. In pleural mesothelioma, local tumor dissemination can occur at chest wall sites following invasive procedures such as biopsies or tube thoracostomy, commonly performed to diagnose the disease or relieve pleural effusion-related dyspnea. Previous studies in pleural mesothelioma patients have reported rates of tumor seeding along procedural tracts based on clinical assessment ranging from 2% to 50%. In this study, we evaluated the pathological incidence, associated risk factors, and prognostic impact of tract involvement in a cohort of 308 patients who underwent pleurectomy decortication with surgical tracts resection. Our findings suggest that malignant tract dissemination is more frequent in epithelioid pleural mesothelioma and is associated with the number of tracts, advanced pathological stage, and decreased overall survival. The association between tract malignant cells dissemination and shorter overall survival suggests that port site involvement may reflect a more aggressive biological phenotype in pleural mesothelioma.

## 1. Introduction

Pleural mesothelioma (PM) is a rare and highly aggressive disease with significant diagnostic challenges. The tumor typically develops along the surfaces of serosal membranes, often mimicking the patterns of various malignancies that metastasize to these sites [1].

Pathological diagnosis of PM primarily relies on histological and immunohistochemical analyses [2]. Diagnosing PM often requires invasive procedures, such as pleural biopsies performed using computer tomography (CT)-guided needles, video-assisted thoracic surgery (VATS), or thoracotomy. The European Society of Thoracic Surgeons (ESTS) recommends obtaining multiple deep tissue biopsies via thoracoscopy to ensure an accurate diagnosis [3]. Similarly, the American Society of Clinical Oncology (ASCO) strongly recommends thoracoscopic biopsies for PM candidates for antineoplastic therapy [4].

While seeding of malignant cells along procedure tracts in patients with secondary pleural cancers is rare, a known complication of these invasive diagnostic or therapeutic procedures in PM is tumor cell seeding along the tracts. Studies have reported tumor seeding through tract rates in patients with PM ranging from 2% to 50% [5]. Because of the risk of tract seeding, the ASCO guidelines recommend minimizing the number of incisions—ideally to two or fewer—and positioning them in locations that can be incorporated into any future definitive surgical resection [4].

In this study, we evaluated patients with PM who underwent resection of prior diagnostic procedural incision sites during pleurectomy decortication (PD) at a high-volume mesothelioma referral center. This is the first study to assess the dissemination of PM cells through prior tracts based on precise pathological evaluation rather than relying on physical examination and clinical assessment of suspected skin nodules. Our objectives were to determine the incidence of disseminated tracts across different PM histological subtypes, identify the associated risk factors, and evaluate the prognosis of patients who develop disseminated PM tracts.

## 2. Materials and Methods

This study is a retrospective review of a prospectively maintained database from a single high-volume center. It included all patients who underwent PD with resection of prior diagnostic sites over a nine-year period (January 2010 to January 2019). The Institutional Review Board (IRB) of the Dana–Farber Cancer Institute (IRB number 98-063) approved the study protocol and data publication. Patient data extracted from the medical records included demographic details, types and reports of diagnostic procedures, neoadjuvant treatment, type of pleurectomy decortication, and pathology findings. Outcome and follow-up data were obtained from outpatient clinic records and from communication with referring physicians or patients. Tumors were staged postoperatively according to the American Joint Committee on Cancer (AJCC) Eighth Edition.

As previously described in our publication [6], the PD procedure involves complete excision of the parietal and visceral pleura and resection of the diaphragm and pericardium if they are macroscopically involved or identified through frozen section analysis. Mediastinal, hilar, intercostal, and internal mammary lymph nodes were sampled. The previous diagnostic biopsy sites were resected and subjected to a pathological evaluation. The resected ports included the skin, subcutaneous tissue, fat, and muscles, extending to the chest wall ribs. Intraoperative heated chemotherapy (IOHC) using cisplatin 175 to 225 mg/m^2^ min lavage at 42.8 °C was circulated for up to 60 min during the procedure.

Resected diagnostic tracts from previously performed biopsies through video-assisted VATS and thoracotomy were classified as positive if tumor cells were identified by a specialized mesothelioma pathologist. Most biopsies were performed using VATS through ports access

Overall survival was calculated from the date of diagnosis and from the PD procedure. Descriptive statistics and cross-tabulations were performed for patients with resected ports. Univariable associations between patient and disease characteristics were assessed using Wilcoxon rank sum and Fisher’s exact tests. Kaplan–Meier estimators were calculated to compare overall survival between groups, and a Cox proportional hazards model was used to evaluate the relationship between overall survival and potential prognostic factors. Two-sided *p*-values of <0.05 were considered significant. Analyses were conducted using R 4.0.3 statistical software.

## 3. Results

Between January 2010 and January 2019, 429 consecutive patients with PM underwent thoracotomy with PD. Among these, 308 patients had prior diagnostic ports used for PM diagnosis included in resection. In this cohort of 308 patients, 233 (75.6%) were men and 187 (61%) underwent right-sided operations, with a median age of 69 years (range, 29–84, mean 67.7). A subset of 67 patients (21.8%) received neoadjuvant therapy. Histological analysis revealed 190 cases (61.7%) with the epithelioid subtype, 108 (35.1%) with the biphasic subtype, and 10 (3.2%) with the sarcomatoid subtype. The median time from the diagnostic port placement to PD resection was 62 days (148 and 52 days in patients with and without neoadjuvant therapy, respectively).

The resected ports primarily consisted of VATS incisions (282 cases), along with 24 thoracotomy incisions. In two cases, the type of diagnostic procedure could not be determined. Among the VATS cases, 116 (41.1%) were uniportal, 96 (34%) involved two ports, 43 (15.3%) had three ports, 15 (5.3%) had four ports, and 12 (4.3%) involved more than four ports. Malignant seeding of the procedural tracts was identified in 69 patients (22.4%). Dissemination with PM cells was observed in 24.7%, 20.4%, and 0% of the epithelioid, biphasic, and sarcomatoid cases, respectively.

Pathological staging based on the American Joint Committee on Cancer (AJCC) Eighth Edition (Table 1) showed the following distribution: T1 (19.8%), T2 (24.4%), T3 (43.5%), and T4 (12.3%). Lymph node involvement was N0 (61.7%), N1 (37.7%), and N2 (0.6%). Overall, patients were staged as 1A (15.6%), 1B (39.0%), 2 (12.3%), 3A (19.2%), 3B (13.6%), and 4 (0.3%).

The median overall survival (calculated from time of diagnosis) was 23.8 months for the entire cohort and 32 months for those with epithelioid histology. The median overall survival (calculated from PD procedure) was 20.5 months for the entire cohort and 27.9 months for those with epithelioid histology. Complete macroscopic resection was achieved in 89% of cases.

Significant differences were noted among the 69 patients with malignant tract seeding (Table 1). Females had a higher rate of tract dissemination than males (32.0% vs. 19.3%, *p* = 0.02). In addition, tract dissemination was significantly correlated with advanced lymph node status (*p* = 0.001) and higher pathological stage (*p* = 0.03) based on the AJCC 8th edition criteria. However, neither neoadjuvant chemotherapy nor T status was significantly associated with tract dissemination. Although dissemination occurred more frequently in epithelioid histology than in non-epithelioid subtypes, the difference was insignificant.

**Figure 1:**
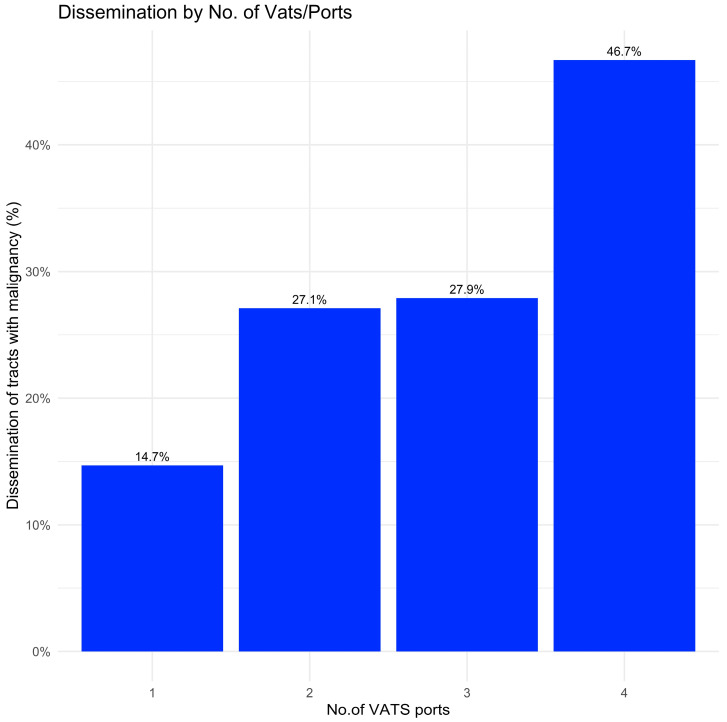
Rate of tract dissemination as a function of the number of VATS ports.

Dissemination rates varied according to the number of VATS ports. Uniportal VATS incisions had significantly lower dissemination rates (14.1%) than VATS with two or more ports (28.3%, *p* = 0.003). Interestingly, the rate of tract dissemination increased to over 40% when using 4-port VATS (Figure 1).

Permanent indwelling drains, such as PleurX, were placed in 40 patients, and tract dissemination was confirmed in six (15%). The dissemination rate in permanent indwelling drains was also significantly higher in females (44.4%) compared to males (6.5%) (*p* = 0.005).

**Figure 2:**
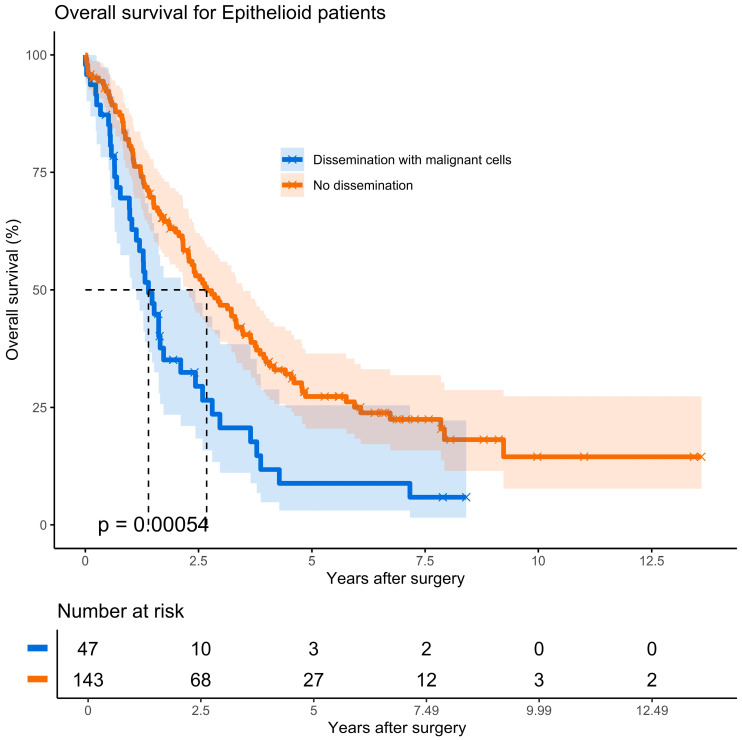
Kaplan–Meier plot depicting estimated survival functions according to port status in epithelioid pleural mesothelioma.

Kaplan–Meier analysis from the time of diagnosis revealed a significant survival difference in 190 patients with epithelioid PM: −36.3 without seeding versus 19.7 months in patients with malignant cells seeding (hazard ratio, 1.9; *p* = 0.001). In this group, the median survival from time of PD was 16.7 months for patients with seeding compared to 32.1 months for those without seeding (Figure 2, hazard ratio, 1.9; *p* < 0.001)

Univariate analysis (Supplementary Table S1) showed factors associated with better overall survival in the 69 patients with disseminated tracts, including early T status, preoperative forced expiratory volume in 1 s (FEV1), early age, administration of intraoperative heated chemotherapy, and adjuvant therapy. Multivariate analysis further confirmed that higher preoperative FEV1, early T status, IOHC, and administration of adjuvant therapy were independently associated with better overall survival in these patients.

## 4. Discussion

This study presents findings from one of the largest known cohorts of patients with PD at a single institution in the modern era. Using data from a prospectively maintained database, we analyzed the incidence, risk factors, and long-term outcomes of tract dissemination after diagnostic procedures for PM in 308 consecutive patients who underwent PD between 2010 and 2019. Resection included prior surgical incisions, with the goal of achieving macroscopic complete resection.

Given PM’s rarity and microscopic similarity to other benign and malignant conditions, establishing a diagnosis can be challenging. The gold standard for a definitive diagnosis is a pleural biopsy performed via thoracoscopy [7]. By providing a direct view of the pleural cavity, mediastinum, and diaphragm, VATS is a valuable tool for evaluating tumor extent [8] and planning surgery. Both the ESMO and NCCN guidelines recommend pleural sampling, especially through thoracoscopy, for patients who have unilateral pleural thickening. To ensure robust subtyping and grading, the ESMO supports sampling biopsies from at least three distant sites [9]. Based on discrepancies we observed between pre- and post-operative diagnoses, our team recently emphasized the vital role of taking multiple thoracoscopic biopsies from different areas of the pleural cavity [10]. We learned from that data that thorough sampling is essential for achieving the correct histology subtype. Therefore, in our clinical practice, we take a minimum of three thoracoscopic biopsies from distinct areas of the parietal pleura. We make sure these biopsies are thorough, extending to the endothoracic fascia. We also routinely use only one port, strategically placed to align with a potential future thoracotomy. This approach is in line with the recent ASCO guidelines, which strongly recommend minimizing the number of incisions and placing them in areas that can be resected during future surgery [4].

Local dissemination of the tumor in PM is a complication that occurs at sites on the chest wall where invasive procedures such as needle biopsy, surgical biopsy either by VATS or thoracotomy, and tube thoracostomy are performed to diagnose or treat common PM symptoms such as dyspnea secondary to accumulation of pleural effusion. The role of prophylactic irradiation of PM tracts has been debatable and controversial during the last three decades, and recommendations from international guidelines have also been inconsistent. Historically, prophylactic irradiation was recommended [11] to reduce the risk of tumor cell seeding based on one of the earliest randomized clinical trials [12], whereas two large recent randomized trials [5,13] demonstrated no advantage with the utility of preventive radiation, although they were criticized for statistical flaws and low power. Interestingly, a meta-analysis and updated systematic review published in 2021 by Lee et al., including all five randomized clinical trials, demonstrated a reduction in tract dissemination using preventive radiation therapy [14]. As of the 2025 update, the American Society of Clinical Oncology (ASCO) does not recommend routine prophylactic radiation to intervention tracts in patients with PM [4].

Here, we report an incidence of dissemination of 22.4%. Importantly, a wide range of malignant dissemination has been reported in the literature (2–50%), with a median value of approximately 20% [15]. In all published studies, identification of tract dissemination was based on physical examination and clinical assessment of palpable nodules in proximity to the pleural intervention sites and not on accurate pathological identification of the malignant PM cells in the resected ports, which was performed in our study. The vast difference seen in the rate of disseminated tracts between old and new clinical trials was explained by Bayman et al. by the possible impact of chemotherapy in reducing tract dissemination in the late studies [5]. Despite this assumption, our study did not observe the effect of neoadjuvant chemotherapy on the rate of tract dissemination with PM. Notably, the dissemination rate in 67 patients who received neoadjuvant chemotherapy was 25.4%.

Factors associated with higher rates of tract dissemination include female sex, advanced N status, number of ports, and pathological stage. Agarwal et al. suggested that the risk of malignant seeding increases with the size of the chest wall incision [16]. In our study, most patients underwent VATS for diagnosis in line with the NCCN, ASCO, and ESMO guidelines. Notably, uniportal VATS was associated with a significantly lower dissemination rate compared to multi-port procedures. The observed link between tract dissemination and reduced survival in epithelioid PM may be explained by its association with more advanced nodal involvement and overall disease stage. This correlation reinforces the notion that port-site dissemination may serve as a marker of more aggressive biological behavior in pleural mesothelioma. Although PM is less common in females, it is more frequently associated with the epithelioid subtype [17,18]. In our study, we observed a higher rate of tract dissemination in epithelioid PM, which may account for the relatively increased dissemination rate in women. Given the small number of affected females (24 with port-site dissemination), the apparent sex-based difference may be exaggerated. This observation is intriguing but remains underexplored and requires validation in larger, multi-institutional cohorts before attributing it to a true biological sex effect.

In one of the few randomized prospective studies by Boutin et al. [12], who assessed the efficacy of radiotherapy in preventing malignant seeding along invasive diagnostic procedures in 40 patients, the experimental group of 20 patients did not develop dissemination through tracts, while the observational group had a 40% occurrence of tract involvement with PM. Although the experimental group had a median survival of 14 months versus only 8 months in the observational group, the difference in survival did not reach statistical significance. In our study, almost 40% of the patients had non-epithelioid histology, and no difference in survival was found in the entire cohort of 308 patients. However, the median overall survival for patients with epithelioid PM who developed tract dissemination as calculated from the date of PD was 16.7 months, which was almost half the length of survival for patients who did not develop this complication (hazard ratio 1.9; *p* = 0.001) (Figure 2). Although the time interval from diagnostic procedures to surgical resection during PD varied, particularly between patients who received neoadjuvant therapy and those who did not, no correlation was found with the rate of port-site dissemination.

Univariate analysis revealed that younger age, higher pre-operative FEV1, early T status, IOHC, and administration of adjuvant therapy were associated with improved overall survival among patients who developed tract dissemination. These factors have previously been identified as favorable prognostic indicators in patients with PM undergoing surgery and are likely not specific to these tract dissemination events. Different research groups have previously demonstrated the role of epithelioid histology in PM as a positive prognostic factor [19,20,21]. However, in the group of 47 patients with epithelioid malignant disseminated tracts, a novel observation revealed that disseminated port in epithelioid PM is associated with a shorter, albeit not statistically significant, overall survival compared to biphasic PM. This finding highlights the negative prognostic impact of malignant dissemination in epithelioid PM.

Given the high incidence of port-site malignant cell dissemination in pleural mesothelioma and the objective of achieving macroscopic complete resection (MCR), we recommend routine port site resection during PD. Our experience shows that prior port resection does not significantly prolong the procedure or increase postoperative complications. Moreover, the association of tract dissemination with poorer prognosis suggests that this subgroup of patients may benefit from consideration of adjuvant chemotherapy or immunotherapy.

## 5. Conclusions

This study is the first to report the incidence of tract dissemination in patients undergoing PD for PM based on the pathological examination of prior incisions. Our findings demonstrated a tract dissemination rate of approximately 25% in patients with PM and revealed novel risk factors for tract dissemination. Tract dissemination is associated with shorter overall survival in patients with epithelioid PM. To alleviate the risk of PM dissemination through incisions and drain tracts, we recommend using a single-port thoracoscopic diagnostic approach and strategically placing the port along a future thoracotomy incision line to ensure its excision during PD. This recommendation is consistent with the updated 2025 ASCO guidelines.

## Figures and Tables

**Table 1:** Patient and disease characteristics.

Variable	Disseminated Ports/69	Non-Disseminated Ports/239	*p*-Value
Age (median, IQR), years	69 (46–83); mean 69	70 (29–84); mean 67.8	0.97
Sex:			**0.02**
Male	45 (19.3%)	188 (80.7%)	
Female	24 (32.0%)	51 (68.0%)	
Histology:			0.15
Epithelioid	47 (24.7%)	143 (75.3%)	
Biphasic	22 (20.4%)	86 (79.6%)	
Sarcomatoid	0 (0%)	10 (100%)	
Laterality:			**0.03**
Rt	34 (18.2%)	153 (81.8%)	
Lt	35 (28.9%)	86 (71.1%)	
Neoadjuvant therapy:			0.51
+	17 (25.4%)	50 (74.6%)	
−	52 (21.6%)	189 (78.4%)	
Type of Incision:			0.1
Thoracotomy	2 (8.3%)	22 (91.7%)	
Thoracoscopy	66 (23.4%)	216 (76.6%)	
VATS ports:			**0.003**
1	18 (14.1%)	110 (85.9%)	
≥2	51 (28.3%)	121 (70.7%)	
T status:			0.43
T1	10 (16.4%)	51 (83.6%)	
T2	15 (20.0%)	60 (80.0%)	
T3	34 (25.4%)	100 (74.6%)	
T4	11 (27.5%)	29 (72.5%)	
Time in days from diagnosis to PD			0.09
Median (IQR)	71 (34–223)	58 (8–255)	
Mean	88.7	78.3	
N status:			**0.001**
N0	32 (16.8%)	158 (83.2%)	
N1	35 (30.2%)	81 (69.8%)	
N2	2 (100%)	0 (0%)	
Pathological stage:			**0.03**
AJCC 8th Edition			
1A	7 (14.6%)	41 (85.4%)	
1B	21 (17.5%)	99 (82.5%)	
2	8 (21.1%)	30 (78.9%)	
3A	20 (33.9%)	39 (66.1%)	
3B	12 (28.6%)	30 (71.4%)	
4	1 (100%)	0	

Bold *p*-values are statistically significant (*p* < 0.05). VATS—Video-assisted thoracoscopic surgery; FEV1—Forced expiratory volume in 1 s; AJCC—American Joint Committee on Cancer.

## Data Availability

The data presented in this study are available upon request from the corresponding author due to privacy and ongoing research.

## Supplementary Materials

The following supporting information can be downloaded at: https://www.mdpi.com/article/10.3390/cancers17172786/s1, Supplementary Table S1: Analysis of association of overall survival in patients with disseminated tracts (n = 69) to patient factors (Cox model).

## Abbreviations

The following abbreviations are used in this manuscript:

PM	Pleural Mesothelioma
PD	Pleurectomy Decortication
VATS	Video-Assisted Thoracic Surgery
AJCC	American Joint Committee on Cancer
