# Supplementary material for: Malignant Local Seeding in Procedure Tracts of Pleural Mesothelioma: Incidence and Novel Risk Factors in 308 Patients
Source: Cancers (Basel). 2025 Aug 26;17(17):2786. doi: 10.3390/cancers17172786 (PMC12427522; doi:10.3390/cancers17172786)
Supplement: Supplementary file 1 [file cancers-17-02786-s001.zip › cancers-3821102-supplementary.pdf]

Supplementary Table S1. Analysis of Association of Overall Survival in Patients with Disseminated Tracts (n=69) to Patients Factors (Cox model)

Variable	Category	Hazard Ratio	P-Value
**Sex**			
	Female	Ref	
	Male	1.26	0.4
**Age, y**			
	≤69	Ref	
	>69	1.81	**0.03**
**Laterality**			
	Right	Ref	
	Left	1.71	0.06
**FEV1**			
	≥80%	Ref	
	<80%	2.04	**0.03**
**N status**			
	N0	Ref	
	N1	1.2	0.57
	N2	2.0	0.35
**T status**			
	1	Ref	
	2	1.17	0.75
	3	2.54	**0.04**
	4	5.93	**0.001**
**Neoadjuvant therapy**			
	-	Ref	0.87
	+	1.05	
**Adjuvant therapy**			
	-	Ref	**0.002**
	+	0.36	
**Histology**			
	Epithelioid	Ref	
	Biphasic	0.77	0.38
**Intraoperative Heated Chemo**	_	Ref	
	+	0.25	**<0.001**

Bold P values are statistically significant(p<0.05)

FEV1-Forced expiratory volume in 1 second
